# Biomechanical phenotypes of unilateral versus bilateral anterior knee pain: evidence from walking kinematics, kinetics, and symmetry indices

**DOI:** 10.3389/fbioe.2026.1867402

**Published:** 2026-07-13

**Authors:** Boyu Zhang, Haojie Wang, Tan Liu, Xu Wang, Pengren Luo, Mengxiang Li, Jiaji Zhang, Jianguo Li, Minghui Zhuang, Chong Lu, Tao Han, Zhefeng Jin, Liguo Zhu

**Affiliations:** 1 The Third Department of Sports Medicine, Wangjing Hospital, China Academy of Chinese Medical Sciences, Beijing, China; 2 Shanxi Hospital of Integrated Traditional and Western Medicine, Taiyuan, Shanxi, China; 3 Beijing Key Laboratory of Orthopedics of Traditional Chinese Medicine, Beijing, China

**Keywords:** anterior knee pain, gait analysis, kinematics, knee extension moment, symmetry index

## Abstract

**Background:**

Anterior knee pain (AKP) is a common musculoskeletal condition with heterogeneous presentations, including unilateral and bilateral symptoms. Although altered gait biomechanics have been reported in AKP, it remains unclear whether unilateral and bilateral AKP exhibit distinct biomechanical phenotypes, particularly in terms of interlimb asymmetry.

**Purpose:**

To compare lower-limb kinematics and joint moments during level walking between individuals with unilateral and bilateral AKP, and to quantify interlimb asymmetry using symmetry indices.

**Methods:**

Seventy-one individuals with AKP (unilateral: n = 36 (16 male and 20 female); bilateral: n = 35 (16 male and 19 female); age range: 11–35) underwent three-dimensional gait analysis at a self-selected speed. Hip, knee, and ankle joint angles (three planes) and normalized joint moments were extracted. The primary kinetic outcome was peak knee extension moment within 0%–60% of the gait cycle. Predefined kinematic and kinetic peak measures for the hip, knee, and ankle joints during defined phases of the stance period were also analyzed. We collected surface electromyographic (sEMG) data from a subset of participants to analyze muscle activation patterns during gait. Interlimb asymmetry was quantified using symmetry indices between more symptomatic (MS) limb for unilateral AKP; and less symptomatic (LS) limb. Between-group comparisons (unilateral vs. bilateral AKP) for baseline characteristics and symmetry indices (SI) were conducted using independent samples t-tests or Mann-Whitney U tests. Within-group differences (more symptomatic vs. less symptomatic limb) for kinematic and kinetic parameters were analyzed using paired t-tests or Wilcoxon signed-rank tests.

**Results:**

Both groups demonstrated significantly reduced peak knee flexion angle and knee extension moment in the more symptomatic limb during stance. Distinct distal adaptations were observed: In the unilateral AKP group, the more symptomatic limb showed lower peak ankle plantarflexion moment (0.95 ± 0.21 N·m/kg vs. 1.06 ± 0.22 N·m/kg, p < 0.05) and a greater foot progression angle (10.03° ± 3.93° vs. 7.62° ± 3.34°, p < 0.05) compared to the less symptomatic limb. In the bilateral AKP group, peak ankle dorsiflexion angle was lower on the more symptomatic side (10.89° ± 3.36°) than on the less symptomatic side (12.60° ± 3.86°) (p < 0.05). Symmetry index analysis revealed no significant between-group differences across most variables, indicating comparable overall asymmetry. sEMG findings showed reduced rectus femoris activation in the symptomatic limb.

**Conclusion:**

Unilateral and bilateral AKP share a protective gait strategy characterized by reduced sagittal-plane knee loading. However, overall gait asymmetry does not differ significantly between groups, suggesting that bilateral AKP may also involve side-specific compensations or globally conservative movement patterns. These findings highlight that the magnitude of interlimb asymmetry alone may not distinguish between unilateral and bilateral presentations. Instead, the observed differences in distal joint adaptations (e.g., ankle dorsiflexion, plantarflexion moment, foot progression angle) provide more informative biomechanical targets for phenotype-specific rehabilitation strategies.

## 1 Introduction

Anterior knee pain (AKP) is a clinical condition encompassing a broad spectrum of etiologies, affecting both athletic populations and the general public. In clinical practice, AKP is often used as an umbrella term to describe symptomatology characterized by peripatellar or retropatellar pain, which is typically exacerbated during weight-bearing functional activities such as walking, stair negotiation, squatting, and running (Duong et al., 2023; Sanchis-Alfonso and Dye, 2017; Gaitonde et al., 2019). However, imaging findings and physical examinations do not consistently correspond to a single, well-defined structural diagnosis.

AKP exhibits notable sex-related differences and represents one of the most prevalent knee disorders, accounting for approximately 25% of all knee injuries (Boling et al., 2010; Glaviano et al., 2015). The reported prevalence of AKP-related conditions is 22.7% in the general population and increases to 28.9% among adolescents (Smith et al., 2018). Females constitute a high-risk group, with a prevalence of approximately 12%–13% in women aged 18–35 years (Slotkin et al., 2018; Roush and Curtis Bay, 2012; Bartsch et al., 2024). These findings underscore the substantial public health burden and clinical significance of AKP.

Current evidence suggests that AKP is a multifactorial condition resulting from the interplay of various factors, including structural alignment, neuromuscular control, load exposure, psychosocial influences, and dynamic movement patterns (Leal et al., 2020; Milovanović et al., 2023). Consistent with this multifactorial nature, AKP is recognized as a heterogeneous condition with considerable variability in clinical presentation, including differences in pain laterality, symptom triggers, and potential underlying mechanisms such as patellofemoral malalignment, muscular imbalance, or soft tissue dysfunction. Collectively, these characteristics indicate that the primary clinical challenge of AKP does not lie in identifying a static structural abnormality, but rather in understanding how joint loading is generated and distributed during functional, weight-bearing activities. Such heterogeneity raises the possibility that distinct AKP subgroups may adopt different movement strategies to unload the painful knee. For instance, individuals with unilateral symptoms may rely more heavily on compensatory mechanisms in the contralateral limb, whereas those with bilateral involvement may exhibit globally conservative movement patterns that limit overall joint loading on both sides. Understanding whether and how these phenotypic differences translate into measurable variations in gait biomechanics is essential for developing targeted, subgroup-specific rehabilitation interventions.

Three-dimensional motion capture–based gait analysis provides an objective method for functional assessment. Walking is a stable and reproducible task that enables evaluation of lower-limb joint loading during the stance phase, including load acceptance, energy transfer, and propulsion, under relatively controlled conditions, thereby offering valuable insights into knee function (Hutchison et al., 2023). By simultaneously capturing kinematic (joint angles) and kinetic (joint moments and ground reaction forces) data, it helps explain the exacerbation of symptoms in individuals with AKP during functional activities. Surface electromyography (sEMG) can be integrated with gait analysis to assess neuromuscular control during walking. sEMG provides direct insight into muscle activation patterns, allowing detection of altered quadriceps activity, delayed onset timing, or reduced activation intensity—all of which have been implicated in AKP pathophysiology and may contribute to altered joint loading and pain perpetuation. Previous studies have shown that individuals with AKP may exhibit altered sagittal-plane knee strategies, such as reduced knee flexion during stance and decreased knee extensor moments, along with compensatory adaptations including altered ankle push-off, foot progression patterns, and differences in frontal-plane hip control (Li et al., 2019; Abdallat et al., 2020; Hag et al., 2021), which are generally considered pain-related protective motor strategies. However, findings remain inconsistent, potentially due to methodological heterogeneity, including variations in inclusion criteria, symptom definitions, and insufficient consideration of pain laterality and the statistical non-independence of bilateral limbs.

Pain laterality represents a key clinical phenotype of AKP with some patients presenting unilateral symptoms and others exhibiting bilateral involvement of varying severity; however, current treatment approaches for different AKP subtypes lack a clear biomechanical basis (Kim and Park, 2022; Chen et al., 2015). The present study aimed to compare lower-limb kinematics and joint moment characteristics during level walking between individuals with unilateral and bilateral AKP using three-dimensional gait analysis, to examine associated neuromuscular patterns via sEMG, and to quantify interlimb asymmetry through symmetry indices. We hypothesized that (1) compared with individuals with bilateral AKP, those with unilateral AKP demonstrate greater interlimb asymmetry in predefined kinematic and kinetic variables; (2) during self-selected walking, interlimb biomechanical differences are significantly correlated with pain laterality.

## 2 Methods

### 2.1 Participants

This retrospective study included patients with knee disorders who attended the Sports Medicine Department (Third Division) of Wangjing Hospital between January and December 2025 and underwent gait analysis. This study protocol was approved by the Ethics Committee of Wangjing Hospital, China Academy of Chinese Medical Sciences (Approval No.: WJEC-KT-2025–064-P001). Data were obtained from the gait laboratory database and electronic medical records. Potential participants were initially identified by searching symptom- and diagnosis-related terms (e.g., anterior knee pain, peripatellar or retropatellar pain, patellofemoral pain, and knee tendinopathy), combined with records of gait analysis, three-dimensional motion capture, and force platform assessments to ensure availability of analyzable gait data.

Inclusion criteria were as follows: (1) patients aged 10–35 years with regular physical activity (≥1 h/day) and a relevant clinical diagnosis; (2) pain provoked by at least two of the following activities: prolonged sitting, stair negotiation, resisted knee extension, squatting, kneeling, running, or jumping; (3) symptom duration between 1 and 4 weeks; and (4) pain intensity ≥3 on the visual analog scale (VAS).

Exclusion criteria included: (1) identifiable structural causes of knee pain (e.g., rheumatic diseases, arthritis, ligament or meniscal injuries, or fractures); (2) patellar dislocation or subluxation; (3) history of lower-limb surgery within the past year; (4) neurological disorders affecting gait; (5) significant lower-limb malalignment (e.g., varus or valgus deformity); and (6) pain localized to the lateral or posterior knee. Demographic characteristics and symptom duration were recorded for all included participants.

From an initial pool of 152 screened candidates, 81 were excluded based on the predefined criteria. Identifiable structural cause of knee pain (rheumatic disease, osteoarthritis, ligament or meniscal injury, fracture): 22 exclusions; Pain due to acute trauma (e.g., fall, direct blow) or symptom duration <1 week or >4 weeks: 18 exclusions; History of knee dislocation/subluxation: 5 exclusions; Lower extremity surgery within the past year: 6 exclusions; Neurological disorder affecting gait: 4 exclusions; Pronounced lower limb malalignment (varus/valgus deformity >5°): 7 exclusions; Pain localized exclusively to lateral or posterior knee (not peripatellar anterior pain): 12 exclusions; Did not meet clinical provocation criteria or VAS <3: 7 exclusions. A total of 71 patients with AKP were enrolled, comprising 36 unilateral and 35 bilateral cases. There were no statistically significant differences between the two groups regarding age (p = 0.47), BMI (p = 0.22), or symptom duration (p = 0.31). Furthermore, walking speed was comparable between groups, showing no significant statistical deviation. The baseline demographic and clinical characteristics of the participants are summarized in Table 1.

**TABLE 1:** Baseline characteristics and spatiotemporal gait parameters.

Basic information		Unilateral AKP		Bilateral AKP	P value
Sex (F/M)		20/16		19/16	—
Age (years)		23.64 ± 9.3		25.17 ± 8.6	0.47
BMI/(kg/m^2^)		21.66 ± 3.9		22.79 ± 3.8	0.22
Walking speed/m·s^-1^		1.06 ± 0.10		1.02 ± 0.10	0.10
Symptom duration/w		2.167 ± 0.87		2.371 ± 0.80	0.31
Step length	more symptomatic limb (m)	1.11 ± 0.11	<0.05	1.09 ± 0.15	<0.05

### 2.2 Gait analysis protocol and quality control

**FIGURE 1:**
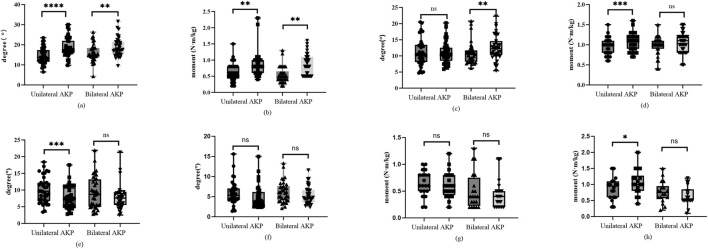
Image shows box plots comparing key gait biomechanical parameters between unilateral and bilateral anterior knee pain (AKP) groups: **(a)** knee flexion angle (°), **(b)** knee extension moment (N·m/kg), **(c)** ankle dorsiflexion angle (°), **(d)** ankle plantarflexion moment (N·m/kg), **(e)** foot progression angle (°), **(f)** hip adduction angle (°), **(g)** hip extension moment (N·m/kg), and **(h)** hip flexion moment (N·m/kg). Data are presented as the median and interquartile range, with individual data points plotted. Between-group comparisons were performed using the independent t-test or Mann–Whitney U test. Statistical differences were defined as ns (P ≥ 0.05), * (P < 0.05), ** (P < 0.01), *** (P < 0.001), and **** (P < 0.0001).

Gait Data Acquisition Gait data were acquired using a BTS optical motion capture system (BTS Bioengineering, Italy) with a sampling frequency of 100 Hz for cameras and 1,000 Hz for force platforms. Reflective markers were placed on specific anatomical landmarks according to the Helen Hayes model, including the acromion, C7, iliac crests, ASIS, greater trochanter, femoral condyles, fibular head, malleoli, metatarsals, and calcaneus. Following a 3-min familiarization period, participants performed 15-m overground walking trials at a self-selected speed. Each participant completed at least three valid walking trials. Select the qualified trial from the three trials conducted. A trial was considered valid if it met all of the following criteria: (1) unambiguous foot contact between the target limb and the force plate; (2) a clearly identifiable heel-strike event in the vertical ground reaction force waveform; (3) a complete, uninterrupted stance phase—defined as the interval from heel-strike to toe-off—for both the left and right feet, with no evidence of gait deviation or intentional force-plate targeting. Among the valid trials, the one exhibiting the highest fidelity to natural gait—i.e., optimal adherence to all validity criteria combined with the smoothest kinematic trajectory in the motion capture data—was selected for analysis. This selection protocol ensured that the analyzed gait cycles accurately reflected each participant’s habitual walking pattern within the constraints of the experimental setup. The software interface is shown in Figure 1.

Data Processing and Normalization Kinematic (joint angles) and kinetic (normalized joint moments) data for the hip, knee, and ankle across three planes were calculated using BTS workstation software. The Helen Hayes model was applied for data processing, and an age-appropriate adult template was selected for all participants. Joint kinetics were normalized to body mass (Nm/kg). Joint moments were derived from inverse dynamics calculations using synchronized ground reaction force data from the force plates, representing external moments acting on each joint.

Key gait events were identified to ensure accuracy, using a multi-source approach:Force Plate Data: The initial contact (heel strike) was defined as the time point when the vertical ground reaction force (GRF) on the corresponding force plate exceeded a 20 N threshold. Toe-off was defined as the time point when the vertical GRF fell below 20 N. This provided objective kinetic criteria.3D Motion Capture Model: The reconstructed 3D marker trajectories and skeleton model within the software (BTS workstation) were visually inspected. The time point of heel marker acceleration (deceleration upon contact) and toe marker liftoff trajectory were cross-referenced to confirm the force plate events. Camera Image Verification: Simultaneous video recordings from the motion capture cameras were reviewed. The video frames corresponding to the force-derived events were examined to visually confirm the precise moment of foot-floor contact and separation, ensuring the absence of marker artifact or foot-slip that could affect the event detection.

The entire gait cycle was then segmented and time-normalized to 0%–100%, with 0% representing the initial contact of the target limb, as determined by the above multi-source method. The primary analysis focused on the stance phase (from initial contact to toe-off) and the late stance/push-off sub-phase, rather than a fixed percentile interval, to better capture functional loading patterns. This event-based approach ensures that the analyzed intervals correspond to biomechanically meaningful phases of gait.

### 2.3 Grouping and side definition

#### 2.3.1 Patient classification

Participants were categorized into two groups based on the clinical presentation of symptoms:

Unilateral AKP Group: Patients with anterior knee pain localized to a single limb, with the contralateral limb documented as asymptomatic.

Bilateral AKP Group: Patients with bilateral symptomatic knee pain.

#### 2.3.2 Symmetry and side definition

To evaluate biomechanical asymmetries, limbs were further classified as more symptomatic (MS) or less symptomatic (LS) within each participant:

In the Unilateral Group: The MS side was the painful limb, while the LS side was the asymptomatic contralateral limb.

In the Bilateral Group: We defined the MS side as the limb exhibiting the higher self-reported visual analog scale (VAS) pain score, provided no confounding factors were present (e.g., acute trauma or recent analgesic use). We designated the contralateral limb—with the lower VAS score—as the LS side.

#### 2.3.3 Variables for descriptive analysis

The primary variables recorded for descriptive analysis included sex, Body Mass Index (BMI), and walking speed.

### 2.4 Outcome measures

Kinematic Variables Joint angle waveforms for the hip, knee, and ankle were extracted across the sagittal, frontal, and transverse planesPeak angles were identified during specific intervals of the stance phase:

Primary Kinematic Outcomes Peak knee flexion during the single-limb support phase (10%–40% of the gait cycle [GC]) and peak ankle dorsiflexion during late stance (40%–60% GC) (Bazett-Jones et al., 2023; Mills et al., 2013; Perry and Burnfield, 2010). Of note, these percentage ranges served as approximate reference intervals; actual peak values were identified based on each participant’s physiologically defined stance phase (heel strike to toe-off), accommodating inter-individual variability in gait timing.

Secondary Kinematic Outcomes Peak hip adduction during the stance phase (approximately 10%–40% GC), likewise identified within the individual’s stance phase rather than a fixed percentage window (Bazett-Jones et al., 2023; Salsich and Long-Rossi, 2010).

Sign Convention Positive values denote flexion, adduction, and dorsiflexion.

Kinetic Variables Joint moments were normalized to body mass (Nm/kg) to account for individual anthropometric differences. To ensure data reliability, only moments with distinct, identifiable peaks within the specified intervals were extracted:

Primary Kinetic Outcomes Peak knee extension moment during mid-stance and peak ankle plantarflexion moment during late stance.

Secondary Kinetic Outcomes Peak hip extension moment during early stance and peak hip flexion moment during late stance.

Sign Convention Positive values denote extension moments.

Spatio-temporal Parameters Step length and walking speed were recorded for descriptive statistics and to characterize the baseline gait profile.

**FIGURE 2:**
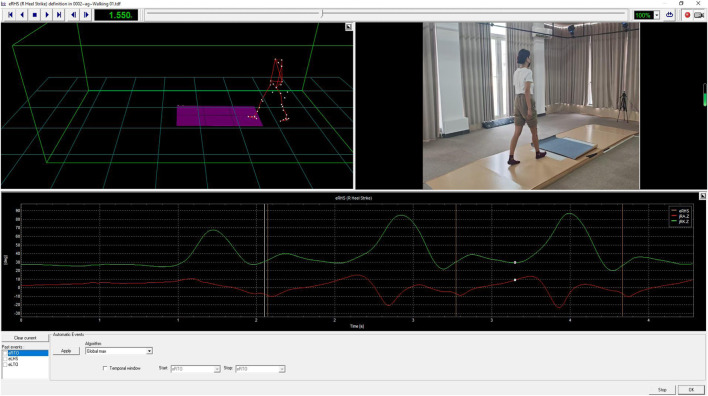
Image shows the demonstration of gait analysis operation.

The intervals and peak values of gait data extraction are shown in Figure 2.

### 2.5 Electromyographic analysis

Surface electromyography (sEMG) data were available for a subset of participants. In the unilateral AKP group, recordings were obtained from the rectus femoris (n = 13), tibialis anterior (n = 19), lateral gastrocnemius (n = 13), and medial gastrocnemius (n = 14); corresponding sample sizes in the bilateral AKP group were 13, 17, 19, and 14, respectively. sEMG signals were recorded synchronously with gait data using the BTS wireless sEMG system (BTS Bioengineering, Italy) at a sampling frequency of 1,000 Hz. Electrodes were placed over the muscle bellies following SENIAM guidelines, with prior skin preparation (shaving and alcohol cleansing). Raw signals were band-pass filtered (20–450 Hz), full-wave rectified, and low-pass filtered (6 Hz) to generate a linear envelope. Muscle activity was recorded during walking for these muscles. For each muscle and each gait cycle, the peak EMG amplitude (μV) was identified during the stance and push-off phases. Peak amplitudes were averaged across valid gait cycles for each participant (Murley et al., 2009; Powers et al., 1996).

### 2.6 Symmetry indices

To quantify interlimb asymmetry, symmetry indices (SI) were calculated for predefined peak kinematic and kinetic variables, comparing the more symptomatic limb with the less symptomatic limb (Robinson et al., 1987). SI was computed using a standardized formula:

$$\text{SI}=100\times \left|X\_\text{more}-X\_\text{less}\right|/\;\left[0.5\times \left(X\_\text{more}+X\_\text{less}\right)\right],$$

where X_more and X_less represent the peak values of the more symptomatic and less symptomatic limbs, respectively. Higher SI values indicate greater asymmetry.

### 2.7 Statistical analysis

Statistical analyses were performed using SPSS version 26.0 (IBM Corp., Armonk, NY, United States of America) and GraphPad Prism version 5.0 (GraphPad Software, San Diego, CA, United States of America). Continuous variables are expressed as mean ± standard deviation (SD). Between-group comparisons of baseline characteristics and symmetry indices (SI) between the unilateral and bilateral AKP groups were conducted using independent samples t-tests for normally distributed data or the Mann-Whitney U test for non-normally distributed data. Within-group comparisons of biomechanical parameters (e.g., between the more symptomatic and less symptomatic limbs) were performed using paired t-tests for normally distributed data or the Wilcoxon signed-rank test for non-normally distributed data. All statistical tests were two-tailed, and the significance level was set at p < 0.05.

## 3 Results

### 3.1 Knee joint: peak knee flexion angle and knee extension moment

During single-limb support in the stance phase, both unilateral and bilateral AKP groups demonstrated significantly lower peak knee flexion angles in the more symptomatic limb compared with the less symptomatic limb (both p < 0.05). Similarly, peak knee extension moments during stance were significantly reduced in the more symptomatic limb in both groups (both p < 0.05). Detailed results are presented in Table 2 and Figure 1.

**TABLE 2:** Peak knee flexion angle and knee extension moment.

Parameter		Unilateral AKP	P value	Bilateral AKP	P value
Knee flexion angle (°)	More symptomatic limb	14.43 ± 4.12	<0.05	16.18 ± 4.59	<0.05
	Less symptomatic limb	18.62 ± 4.17		18.91 ± 4.71	
Knee extension moment (N·m/kg)	More symptomatic limb	0.61 ± 0.28	<0.05	0.53 ± 0.25	<0.05
	Less symptomatic limb	0.86 ± 0.38		0.85 ± 0.32	

### 3.2 Ankle joint: peak dorsiflexion angle, foot progression angle, and ankle moment

During late stance (40%–60% of the gait cycle), the bilateral AKP group demonstrated a significantly lower peak ankle dorsiflexion angle in the more symptomatic limb compared with the less symptomatic limb (p < 0.05), whereas no significant interlimb difference was observed in the unilateral AKP group (p = 0.99). In terms of kinetics, the unilateral AKP group exhibited a significantly higher peak ankle moment in the less symptomatic limb than in the more symptomatic limb (p < 0.05), while no significant difference was found in the bilateral AKP group (p = 0.06). Additionally, the unilateral AKP group showed a significantly greater foot progression angle in the more symptomatic limb (p < 0.05), whereas no significant interlimb difference was observed in the bilateral AKP group (p = 0.11). Detailed results are presented in Table 3 and Figure 1.

**TABLE 3:** Peak ankle kinematics and kinetics.

Parameter		Unilateral AKP	P value	Bilateral AKP	P value
Ankle dorsiflexion angle (°)	More symptomatic limb	11.06 ± 4.26	0.99	10.89 ± 3.36	<0.05
	Less symptomatic limb	11.07 ± 3.42		12.60 ± 3.86	
Ankle plantarflexion moment (N·m/kg)	More symptomatic limb	0.95 ± 0.21	<0.05	0.98 ± 0.23	0.06
	Less symptomatic limb	1.06 ± 0.22		1.05 ± 0.24	
Foot progression angle (°)	More symptomatic limb	10.03 ± 3.93	<0.05	9.21 ± 4.85	0.11
	Less symptomatic limb	7.62 ± 3.34		7.80 ± 3.72	

### 3.3 Hip joint: peak hip adduction angle and hip moments

During the stance phase (10%–40% of the gait cycle), no significant interlimb differences were observed in peak hip adduction angle in either the unilateral or bilateral AKP groups (p = 0.11 and p = 0.13, respectively). In terms of kinetics, peak hip extension moments showed no significant side-to-side differences in either group (unilateral AKP: p = 0.61; bilateral AKP: p = 0.08). However, for peak hip flexion moment, the unilateral AKP group demonstrated a significantly lower value in the more symptomatic limb compared with the less symptomatic limb (p < 0.05), whereas no significant interlimb difference was observed in the bilateral AKP group (p = 0.18). Detailed results are presented in Table 4 and Figure 1.

**TABLE 4:** Peak hip kinematics and kinetics.

Parameter		Unilateral AKP	P value	Bilateral AKP	P value
Hip adduction angle (°)	More symptomatic limb	5.88 ± 2.92	0.11	6.17 ± 2.62	0.13
	Less symptomatic limb	4.94 ± 2.88		5.43 ± 2.23	
Hip extension moment (N·m/kg)	More symptomatic limb	0.64 ± 0.23	0.61	0.51 ± 0.33	0.08
	Less symptomatic limb	0.61 ± 0.25		0.44 ± 0.27	
Hip extension moment (N·m/kg)	More symptomatic limb	0.88 ± 0.35	<0.05	0.77 ± 0.33	0.18
	Less symptomatic limb	1.07 ± 0.39		0.65 ± 0.30	

### 3.4 Surface electromyography results

In the sEMG subset, rectus femoris EMG amplitude was significantly lower in the more symptomatic limb than in the less symptomatic limb in both unilateral and bilateral AKP groups (both p < 0.05). For the tibialis anterior, the unilateral AKP group showed a significantly higher EMG amplitude in the more symptomatic limb (p < 0.05), whereas no significant interlimb difference was observed in the bilateral AKP group (p = 0.37). No significant side-to-side differences were found for the lateral or medial gastrocnemius in either group (all p > 0.05). Detailed results are presented in Table 5.

**TABLE 5:** Peak surface electromyography (EMG) amplitude.

Variable (μV)		Unilateral AKP	P value	Bilateral AKP	P value
Rectus femoris	More symptomatic limb	6.23 ± 2.71	<0.05	4.85 ± 2.64	<0.05
	Less symptomatic limb	10.77 ± 3.98		6.15 ± 3.29	
Tibialis anterior	More symptomatic limb	10.16 ± 3.82	<0.05	9.18 ± 5.15	0.37
	Less symptomatic limb	8.84 ± 4.85		8.23 ± 4.25	
Lateral gastrocnemius	More symptomatic limb	12.62 ± 4.09	0.87	11.58 ± 4.50	0.17
	Less symptomatic limb	12.38 ± 3.81		10.32 ± 4.60	

### 3.5 Symmetry indices (SI): between-group comparison (unilateral vs. bilateral AKP)

No significant between-group differences were observed in symmetry indices for knee flexion angle, knee extension moment, ankle dorsiflexion angle, ankle plantarflexion moment, foot progression angle, hip adduction angle, hip extension moment, or hip flexion moment (all p > 0.05). A trend toward significance was noted for the knee extension moment SI (unilateral: 10.48 ± 7.31; bilateral: 12.35 ± 8.62; p = 0.06). Detailed values are presented in Table 6.

**TABLE 6:** Symmetry indices (SI) of kinematic and kinetic variables.

Parameter	Unilateral AKP	Bilateral AKP	P Value
Knee flexion angle (°)	7.08 ± 4.97	6.62 ± 5.04	0.69
Knee extension moment (N·m/kg)	10.48 ± 7.31	12.35 ± 8.62	0.06
Ankle dorsiflexion angle (°)	7.59 ± 5.06	6.41 ± 4.67	0.37
Ankle plantarflexion moment (N·m/kg)	4.56 ± 2.65	4.85 ± 2.81	0.65
Foot progression angle (°)	8.68 ± 8.05	10.84 ± 6.79	0.22
Hip adduction angle (°)	12.69 ± 8.26	10.45 ± 6.93	0.22
Hip extension moment (N·m/kg)	6.88 ± 4.23	7.69 ± 5.55	0.61
Hip flexion moment (N·m/kg)	8.85 ± 5.45	9.98 ± 6.27	0.56

## 4 Discussion

This study compared interlimb differences and asymmetry during level walking between individuals with unilateral and bilateral anterior knee pain (AKP). Both groups demonstrated reduced peak knee flexion angle and knee extension moment in the more symptomatic limb during stance, indicating a shared protective gait strategy aimed at reducing patellofemoral joint loading. Distal adaptations differed according to pain laterality: bilateral AKP exhibited reduced ankle dorsiflexion in the more symptomatic limb, whereas unilateral AKP showed decreased ankle plantarflexion moment and increased foot progression angle. Notably, symmetry index analysis revealed no consistent between-group differences in overall gait asymmetry. In the sEMG subset, reduced rectus femoris activation in the symptomatic limb was observed, consistent with the decreased knee extension moment and suggesting a potential neuromuscular contribution to these biomechanical alterations.

Both unilateral and bilateral AKP groups demonstrated a consistent pain-avoidance gait strategy, characterized by reduced knee flexion angle and knee extension moment during stance. Reduced knee flexion decreases quadriceps demand, thereby lowering patellofemoral joint reaction forces and stress, which is consistent with previous reports of stiff-knee gait, reduced sagittal-plane work, and decreased knee extensor moments in AKP/PFP populations (Besier et al., 2009; Nunes et al., 2019). This pattern likely reflects a combined effect of pain avoidance and load redistribution, whereby individuals reduce sagittal-plane knee demand to achieve short-term symptom relief (Gallina et al., 2024), potentially at the expense of gait efficiency and increased loading at other joints (Bhatt and Shukla, 2022). Notably, given the short symptom duration in this cohort (1–4 weeks), such protective adaptations may emerge early, highlighting their relevance for early assessment and intervention.

From a neuromuscular perspective, reduced quadriceps activation may contribute to the observed decrease in knee extension moment. In the sEMG subset, rectus femoris activation was lower in the symptomatic limb, consistent with the kinetic findings, supporting quadriceps inhibition or disuse as a potential mechanism underlying altered knee joint loading (Rathleff et al., 2013; Rathleff et al., 2016). Pain-related arthrogenic muscle inhibition and central motor adaptations have been widely reported in AKP/PFP (Cowan et al., 2001; Souza and Powers, 2009; Ferber et al., 2011), and the present findings extend this evidence to walking. However, these results should be interpreted with caution, as sEMG analysis was limited to a subset of muscles and did not capture activation timing. Given the importance of coordinated activation within the quadriceps (e.g., vastus medialis and lateralis) and early stance neuromuscular control for load attenuation, future studies incorporating comprehensive muscle monitoring and phase-specific activation analysis are warranted to better elucidate the relationship between muscle activation, joint moments, and patellofemoral joint loading.

Alterations in the ankle-foot and hip complexes likely reflect multi-joint load redistribution. Distally, these adjustments differ by group: the reduced peak ankle dorsiflexion on the more symptomatic (MS) side in bilateral AKP may alter stance mechanics by restricting anterior tibial translation (Toyooka et al., 2025). Conversely, in unilateral AKP, the decreased ankle plantarflexion moment on the MS side and concurrent increase on the less symptomatic (LS) side suggest that the contralateral limb assumes greater propulsive or supportive demands (Wang et al., 2022). Furthermore, the asymmetric foot progression angle (FPA) in the unilateral group indicates that foot orientation may help modulate lower limb alignment and loading pathways (Azzi et al., 2017).

Proximally, despite non-significant asymmetries in hip adduction angle and extension moment, the inter-limb difference in hip flexion moment in unilateral AKP suggests a side-specific sagittal strategy. Notably, due to data instability, our hip kinetic analysis was restricted to the sagittal plane. This limitation may inadequately capture the coronal and transverse proximal control features frequently emphasized in PFP literature (Kingston et al., 2020). Therefore, conclusions regarding proximal compensation should be interpreted with caution. Future studies will incorporate variables such as hip abduction moments to establish a comprehensive explanatory framework for these mechanisms.

The comparison between unilateral and bilateral AKP challenges the common assumption that unilateral pain is associated with greater interlimb asymmetry. Symmetry index analyses did not demonstrate consistently higher asymmetry in unilateral AKP, with only a trend observed for the knee extension moment. These findings suggest that bilateral AKP does not necessarily reflect a more symmetrical pattern, as functional differentiation between limbs may still exist, and bilateral cases may adopt a globally conservative gait strategy with reduced overall output (Assa et al., 2013), thereby limiting detectable asymmetry. Walking speed may represent an additional confounding factor, as it has systematic effects on joint kinematics (Kirtley et al., 1985; Wade et al., 2022) and kinetics (Buddhadev et al., 2020; Van Hamme et al., 2015), and the lack of speed control in the unilateral group may have influenced between-group SI comparisons. Future studies should incorporate speed-adjusted statistical models (e.g., linear, generalized linear, or mixed-effects models) or apply speed-matching designs to improve causal interpretation.

Clinically, the present findings highlight a consistent reduction in sagittal-plane knee loading in the symptomatic limb, indicating that peak knee flexion angle and knee extension moment may serve as interpretable markers for identifying protective gait patterns and monitoring rehabilitation outcomes. Importantly, differences between unilateral and bilateral AKP appear to be driven more by individualized multi-joint load redistribution than by global asymmetry magnitude, particularly involving distal adjustments such as foot progression and ankle output. Therefore, rehabilitation strategies should extend beyond the symptomatic knee to include contralateral and distal joint function, with attention to potential compensatory overload. Given the strong influence of walking speed on gait biomechanics, it should be routinely recorded and controlled in both clinical and research settings.

## 5 Limitations and future directions

This study has several limitations. The cohort included participants across a wide age range (10–35 years), encompassing prepubertal, pubertal, and adult individuals. Given that anthropometric characteristics, neuromuscular function, and gait biomechanics undergo significant developmental changes during this period, grouping all participants together may introduce heterogeneity and potentially confound the results. As a single-center retrospective analysis, causal inference cannot be established, and selection and information bias may be present. Second, walking was performed at a self-selected speed; although no significant between-group differences were observed, gait speed systematically influences joint kinematics and kinetics and may have confounded interlimb differences and symmetry index comparisons. Limited number of valid gait cycles may reduce the reliability and representativeness of peak measures and SI, while increasing the influence of inter-individual variability. Lack of an asymptomatic age- and gender-matched control group limits the ability to definitively attribute the observed biomechanical patterns specifically to AKP, as we cannot establish a baseline normative gait profile for comparison. This necessitates a degree of inference when discussing adaptive versus pathological mechanisms. Finally, sEMG data were available only in a subset of participants; therefore, the relationship between reduced quadriceps activation and decreased knee extension moment should be interpreted with caution and requires further validation.

## 6 Conclusion

Both unilateral and bilateral AKP exhibited reduced peak knee flexion angle and knee extension moment in the more symptomatic limb during walking, supporting a shared gait strategy aimed at reducing patellofemoral joint loading. Pain laterality (unilateral vs. bilateral) did not result in a clear separation of symmetry indices, suggesting that bilateral AKP may also involve side-specific compensations and load redistribution. Future studies should further validate gait characteristics across AKP phenotypes by incorporating speed-controlled designs, precise phase-specific analyses, and more comprehensive assessments of proximal kinetics and neuromuscular function.

## Data Availability

The raw data supporting the conclusions of this article will be made available by the authors, without undue reservation.
